# Quality of patient safety indicators in intensive care units: Protocol for a systematic review

**DOI:** 10.1371/journal.pone.0349015

**Published:** 2026-05-26

**Authors:** Isac Davidson Santiago Fernandes Pimenta, Ádala Nayana de Sousa Mata, Gidyenne Christine Bandeira Silva de Medeiros, Daniel Guillén-Martínez, Manuel Pardo Ríos, Grasiela Piuvezam

**Affiliations:** 1 Post-Graduation Program of Public Health, Federal University of Rio Grande do Norte, Natal, Rio Grande do Norte, Brasil; 2 Systematic Review and Meta-analysis Laboratory – Lab-SYS Research & Health Innovation (CNPq – UFRN); 3 Multicampi School of Medical Sciences, Federal University of Rio Grande do Norte, Caicó, Rio Grande do Norte, Brazil; 4 Catholic University of Murcia, Murcia, Spain; University of Porto Faculty of Medicine: Universidade do Porto Faculdade de Medicina, PORTUGAL

## Abstract

Patient safety is an important issue in intensive care units. Patient safety indicators are often incorporated without undergoing the proper process of development and validation, which leads to problems with construct validity, measurement reliability, feasibility, and comparability across institutions and countries. This study reports a protocol for a systematic review that aims to identify and assess the validity of patient safety indicators used in the intensive care unit context. This protocol was previously registered in PROSPERO (CRD42024617125). We will search for original studies in the following databases: PubMed (Medline), Scopus, EMBASE (Elsevier), CINHAL (EBSCO), Web of Science, Cochrane Database and Google Scholar for grey literature. We will include methodological or observational studies that report the construction and/or validation of patient safety indicators. Two reviewers will independently screen and assess the studies, and disagreements will be resolved by a third reviewer. We will assess the quality of the studies using the COnsensus-based Standards for the selection of health status Measurement INstruments (COSMIN) checklist. To assess indicator validity, we will use the Appraisal of Indicators through Research and Evaluation (AIRE) instrument. We will classify the indicators according to Donabedian attributes of structure, process and outcomes, as well as the contexts of intensive care, such as pediatric, neonatal or cardiac units, for example. We will also perform descriptive analysis of each category assessed. We expect that our review will contribute to providing a valid set of indicators and help to identify gaps regarding patient safety dimensions that need to be assessed by new indicators.

## Introduction

Patient safety in healthcare organizations is defined as “a framework of organized activities that creates cultures, processes, procedures, behaviors, technologies and environments in health care that consistently and sustainably lower risks, reduce the occurrence of avoidable harm, make errors less likely and reduce the impact of harm when it does occur” [1].

In intensive care units (ICUs), patient safety activities are among the most important attributes for delivering high-quality care. Patients in ICUs are often highly vulnerable and in critical condition, relying primarily on the work of the staff to support life. Even so, preventable patient safety incidents occur in about 18% of ICU admissions [2].

A key task in achieving patient safety in the ICU context is the measurement of performance. In this sense, many indicator sets have been developed over the last two decades to help governments and healthcare organizations with establishing a benchmark, identify areas for improvement and compare patient safety across countries and institutions [3].

More than 20 years ago, Pronovost et al. [3], developed and pilot tested one of the most acknowledged sets of indicators to measure quality of care and patient safety in ICUs. Their validation study was conducted in 13 ICUs, with 19 indicators. They estimated that, in units with a 1,000 admissions per year, receiving adequate care could reduce one death every two weeks and up to 1,800 extra days in ICU and hospital care, highlighting the importance of quality and safety monitoring. Later, other organizations also developed their own sets, such as the Agency for Healthcare Research and Quality (AHRQ), Joint Commission on Accreditation of Healthcare Organizations and European Society of Intensive Care [4–6].

As a quality dimension assessment, patient safety indicators (PSI) can be categorized according to Donabedian’s attributes: structure – the attributes of the service such as staff, infrastructure, etc; process – the way the service processes work to deliver care; and outcomes – the impact on the patient resulting from the work set [7,8]. Some indicators, such as patient-to-nurse ratio (structure), thromboprophylaxis in ventilated patients (process), mortality rates, and length of stay (outcomes), are widely used to assess ICU care and are often collected based on patient records, which are considered the gold standard source of information for these indicators [5].

Despite the importance of PSI in improving patient safety and quality of care, some questions arise regarding their use. The first is related to construct validity. Defining what is important to measure is a challenge for healthcare organizations. It is necessary to specify patient safety goals and reflect on whether they are aligned with another quality dimension and what will be prioritized. A patient safety goal may not be an efficiency goal, for example. Therefore, it is important to define what matters to patients and organizations [9,10].

Another issue is feasibility. The size of the set of indicators and the method of data collection must be considered in performance assessment. Having too many indicators could place a burden on healthcare professionals, especially nurses, and be highly costly for healthcare organizations [9]. Some studies indicate that an average of 8.5 days per month can be spent manually collecting data for a small set of indicators [11,12]. On the other hand, too few indicators may not provide sufficient information to assess the safety of care [9].

Another point to consider is that many indicators focus primarily on outcome measures, such as mortality, length of stay, readmissions and incidence of infections. However, it is also necessary to assess process and structure measures, considering the need for improvement and the logical correlation between these dimensions in order to achieve better outcomes [13,14].

Finally, many institutions create and adopt PSI sets but do not analyze their reliability or the influence of pre-existing conditions, which are necessary to distinguish incidents apart from complications arising during care [6,15–17]. The quality of patient records may also influence the detection of incidents and introduce heterogeneity to the measurements [6,18].

Considering this, it is necessary to identify and assess the PSI available for evaluating performance in ICUs, in order to provide a set of valid and reliable indicators that can help healthcare providers improve performance and patient safety. Therefore, we report in this paper a protocol for a systematic review that aims to identify and assess the validity of patient safety indicators used in the ICU context.

## Methods and analysis

### Study registration and reporting

This protocol was previously registered in the International Prospective Register of Systematic Reviews (PROSPERO) under the code CRD42024617125. This report is based on the recommendations of the Preferred Reporting Items for Systematic Reviews and Meta-Analyses Protocols (PRISMA-P) statement (S1 File) and the Cochrane Handbook for Systematic Reviews [19,20].

### Eligibility criteria

#### Construct of Interest.

Indicators that quantitatively measure patient safety, defined as “the absence of preventable harm to a patient and reduction of risk of unnecessary harm associated with health care to an acceptable minimum”. This includes, but is not limited to, measures used for monitoring or assessing patient safety, such as questionnaires, standards, criteria, norms or scales.

#### Type of Participants.

Critical care patients and professionals (physicians, nurses, physiotherapists, and others).

#### Type of Outcomes.

We will consider the following outcomes in the included studies:

Indicator description: name of the indicator, definition, coverage (population and period of observation), formula for calculation (numerator and denominator), data source, exceptions to the indicator, missing data protocol, risk adjustments (by health condition, social determinants and others), benchmarking and references.

Indicator validity: We will consider the reporting of processes to achieve content validity, scientific evidence support, relevance to the organizational context and stakeholder involvement.

Measurement validity: We will consider measures of reliability and feasibility (test, retest), responsiveness, viability and interpretability of the indicators.

#### Study types.

Studies that report the development and validation of patient safety indicators. This may include validation studies using consensus methods (such as Delphi panels, consensus conferences, focus groups, RAND/UCLA appropriateness method and others), observational studies (cohorts, cross-sectional) or mixed-methods studies.

#### Exclusion Criteria.

We will exclude indicators: related to transportation to ICUs or other units; and indicators related to palliative/end-of-life care. Studies that do not provide a description of the indicators will also be excluded.

### Search strategy and information sources

We will search for studies in the following databases: PubMed (Medline), Scopus, EMBASE (Elsevier), CINHAL (EBSCO), Web of Science and Cochrane Database. We will also search for grey literature in Google Scholar. For our search we will combine MeSH and Emtree terms according to each database and include relevant non-index terms. We present in the supplementary file (S2 File) a search equation for the PubMed (Medline) database. There will not be any restrictions on publication years or language. In case we found a study written in other languages than Portuguese, Spanish and English, we will seek assistance from the language center of our university to support the translation of the study.

### Study selection

After extracting the records from the databases, we will proceed with identifying and removing duplicates. Two independent reviewers will then screen the records assessing the titles and abstracts. The selected studies will be read in full to assess their eligibility according to the inclusion criteria. We will also screen the reference lists of the included studies to identify additional relevant studies not captured in the main search. Disagreements between reviewers will be resolved by consensus or, if necessary, by a third reviewer. We will use the software Rayyan® to perform all the stages of study selection.

### Data extraction and management

Two reviewers will independently extract the following data: study identification (main author, year of publication, country, type of critical care unit); population characteristics (age, sex, profession); indicators specifications (description, formula – numerator and denominator; exclusions, data source, period, evidence support, benchmark); type of validation; and indicator proprieties (content validity index, results of correlation tests, results of factorial analysis, pre-test results). We will use a previously tested spreadsheet to collect the data.

#### Dealing with missing data.

In case of missing or unclear data, we will attempt to contact the corresponding author of the study by e-mail. If we do not receive a response, the data will be excluded from the analysis, and this will be reported in the review. We will also discuss the potential impact of missing data on the interpretation of the results in the discussion section.

### Quality assessment

To assess the methodological quality of the studies, two independent reviewers will use the COnsensus-based Standards for the selection of health status Measurement INstruments (COSMIN) Risk of Bias checklist. The COSMIN checklist is organized into 10 domains, each focusing on a different measurement property or methodological aspect. Each domain is used only if applicable to the study and includes standards and items that are rated on a four-point scale (very good, adequate, doubtful and inadequate). The COSMIN checklist uses the principle of ‘worst score counts’, indicating that for each domain, the lowest rating determines the overall rating of the domain [21].

To assess indicator validity, we will perform the quality assessment of the indicators using the Appraisal of Indicators through Research and Evaluation (AIRE) instrument [22]. AIRE assesses the indicators in 20 items divided into four domains: purpose, relevance and organizational context; stakeholder involvement; scientific evidence; and additional evidence, formulation and usage. Each item is scored from 1 (strongly disagree) to 4 (strongly agree). Two reviewers will independently assess the set of indicators. For each domain, total scores will be calculated by summing the individual reviewers’ scores and standardizing the total as a percentage of the maximum possible score for that domain.

### Data synthesis

We will conduct a narrative analysis of all PSI. The indicators extracted from the studies will be first checked to eliminate redundancy. After that, they will be classified in Donabedian attributes of structure, process and outcomes.

The indicators will also be categorized based on whether they are applicable to all intensive care contexts or to specific care settings, such as pediatric, neonatal, or cardiac units, for example. We will perform descriptive analysis of each assessed category using counts and proportions. At the end, we will produce an inventory of PSI categorized by their characteristics and quality, presented in a summary table. Given the outcomes of our review, we will not conduct a quantitative synthesis of the data.

### Dissemination and ethics

The final review will be published in a peer-review scientific journal and may also be presented in abstracts for poster or oral presentations. We also plan to disseminate the results to national and international stakeholders, including healthcare organizations, professionals, and patients. As this review will use secondary data only, ethical approval from our institution will not be required.

## Discussion

Patient safety is a key feature of ICU care. The literature points out that the incidence of adverse events in ICU is three times higher than in general hospital care [2]. Therefore, it is necessary to monitor and assess patient safety through PSI, creating information for healthcare providers and organizations to correctly identify improvement opportunities and evaluate the impact of quality improvement projects [18].

Still, it is very common in healthcare services to create or incorporate indicators that have not undergone a validation process, resulting in non-standardized data collection and analysis procedures. These often become a burden for professionals and patient safety or quality offices [10].

The variation in the PSI sets across organizations and even countries also makes it very difficult to compare results and estimate the burden of patient safety issues in ICUs, making the development of broader initiatives to improve patient safety in this setting a challenge [6].

In addition, current systematic reviews have some limitations. Some focus only on quality indicators related to specific patient conditions, such as palliative care or cardiovascular care [23–25]. Other studies that address quality indicators in ICUS do not explore indicator and measurement validity, and their searches are limited to the Pubmed/Medline database, which restricts access to relevant studies [14,26]. Therefore, it is necessary to identify, through a systematic review, reliable PSIs for use in ICU care.

### Limitations

It is important to identify and acknowledge some limitations. The review will not provide a general prevalence estimate of adverse events, their causes, or their relationship with demographic characteristics.

Although our search strategy includes no restrictions on language and our research team is proficient in Spanish, Portuguese, and English, there remains a potential for language bias. This is primarily due to the indexing limitations of some databases, which may underrepresent studies published in other languages or fail to provide adequate metadata for non-English publications. To mitigate this, we will include grey literature and manually screen reference lists. Additionally, we benefit from institutional support through our university’s language center, which assists with translation and interpretation of studies published in other languages.

Despite these efforts, relevant research in languages beyond our team’s and institution’s capabilities may still be missed, and this limitation should be considered when interpreting the comprehensiveness of our findings.

It is also important to consider that this review will focus on evaluating the methodological quality of measurement tools rather than assessing the effectiveness of their implementation in clinical practice.

Nevertheless, we believe our review will provide a valid set of patient safety indicators that can support healthcare organizations in developing programs to improve patient safety. Additionally, our review will help identify gaps in existing PSIs, highlighting priorities for the development of new indicators.

## Supporting information

S1 File PRISMA-P checklist. (DOC)

S2 File Preliminary search strategy for Pubmed/Medline. (DOCX)
