# Supplementary material for: Quality of patient safety indicators in intensive care units: Protocol for a systematic review
Source: PLoS One. 2026 May 26;21(5):e0349015. doi: 10.1371/journal.pone.0349015 (PMC13210177; doi:10.1371/journal.pone.0349015)
Supplement: S2 File — (DOCX) [file pone.0349015.s002.docx]

**S2 File. Search strategy for Pubmed/Medline**

#1 ("Critical Care"[Mesh]) OR (Care, Critical) OR (Intensive Care) OR (Care, Intensive)

#2 ("Intensive Care Units"[Mesh]) OR (Intensive Care Unit) OR (Unit, Intensive Care) OR (ICU Intensive Care Units)

#3 = #1 OR #2

#4 ("Patient Safety"[Mesh]) OR (Patient Safeties) OR (Safety, Patient)

#5 ("Patient Harm"[Mesh]) OR (Patient Harm)

#6 ("Patient Harm"[Mesh]) OR (Harms, Patient)

#7 ("Iatrogenic Disease"[Mesh]) OR (Disease, Iatrogenic) OR (Diseases, Iatrogenic) OR (Iatrogenic Diseases) OR (Hospital-Acquired Condition) OR (Condition, Hospital-Acquired) OR (Conditions, Hospital-Acquired) OR (Hospital Acquired Condition) OR (Hospital-Acquired Conditions)

#8 ("Medical Errors"[Mesh]) OR (Errors, Medical) OR (Error, Medical) OR (Medical Error) OR (Medical Mistake) OR (Mistake, Medical) OR (Mistakes, Medical) OR (Medical Mistakes) OR (Medical Errors of Omission) OR (Omission Medical Error) OR (Omission Medical Errors) OR (Medical Error of Omission) OR (Medical Errors of Commission) OR (Medical Error of Commission) OR (Commission Medical Error) OR (Commission Medical Errors) OR (Critical Medical Incidents) OR (Critical Medical Incident) OR (Incident, Critical Medical) OR (Incidents, Critical Medical) OR (Medical Incident, Critical) OR (Medical Incidents, Critical) OR (Critical Incidents, Medical) OR (Critical Incident, Medical) OR (Incident, Medical Critical) OR (Incidents, Medical Critical) OR (Medical Critical Incident) OR (Medical Critical Incidents) OR (Wrong-Patient Surgery) OR (Surgeries, Wrong-Patient) OR (Surgery, Wrong-Patient) OR (Wrong-Patient Surgeries) OR (Wrong Patient Surgery) OR (Wrong-Procedure Errors) OR (Errors, Wrong-Procedure) OR (Error, Wrong-Procedure) OR (Wrong-Procedure Error) OR (Wrong Procedure Errors) OR (Wrong-Site Surgery) OR (Surgeries, Wrong-Site) OR (Surgery, Wrong-Site) OR (Wrong-Site Surgeries) OR (Wrong Site Surgery) OR (Surgical Errors) OR (Errors, Surgical) OR (Error, Surgical) OR (Surgical Error) OR (Never Event) OR (Event, Never) OR (Events, Never) OR (Never Events)

#9 = #4 OR #5 OR #6 OR #7 OR #8

#10 ("Quality Indicators, Health Care"[Mesh]) OR (Quality Indicators, Healthcare) OR (Healthcare Quality Indicator) OR (Healthcare Quality Indicators) OR (Indicator, Healthcare Quality) OR (Indicators, Healthcare Quality) OR (Quality Indicator, Healthcare) OR (Global Trigger Tool, Healthcare) OR (Healthcare Global Trigger Tool) OR (Health Metrics) OR (Health Metric) OR (Metrics, Health)

#11 = #3 AND #9 AND #10
